# Thermodynamic Analysis of Financial Markets: Measuring Order Book Dynamics with Temperature and Entropy

**DOI:** 10.3390/e26010024

**Published:** 2023-12-25

**Authors:** Haochen Li, Yue Xiao, Maria Polukarov, Carmine Ventre

**Affiliations:** Department of Informatics, King’s College London, Bush House, Strand, London WC2R 2LS, UK; haochen_li@kcl.ac.uk (H.L.); yue.1.xiao@kcl.ac.uk (Y.X.); maria.polukarov@kcl.ac.uk (M.P.)

**Keywords:** econophysics, market temperature, market entropy, liquidity, volatility, limit order book, market microstructure, cryptocurrency markets

## Abstract

This study bridges finance and physics by applying thermodynamic concepts to model the limit order book (LOB) with high-frequency trading data on the Bitcoin spot. We derive the measures of Market Temperature and Market Entropy from the kinetic and potential energies in the LOB to provide a deeper understanding of order activities and market participant behavior. Market Temperature emerges as a robust indicator of market liquidity, correlating with liquidity measures such as Active Quote Volume, bid–ask spread and match volume. Market Entropy, on the other hand, quantifies the degree of disorder or randomness in the LOB, providing insights into the instantaneous volatility of price in the high-frequency trading market. Our empirical findings not only broaden the theoretical framework of econophysics but also enhance comprehensive understanding of the market microstructure and order book dynamics.

## 1. Introduction

This study investigates the intricate dynamics of the limit order book (LOB) through a novel econophysics approach. This interdisciplinary approach is based on the application of thermodynamic principles, particularly the concepts of temperature and entropy, to reveal and understand the complex behaviors of the market participant and their order activities in the financial markets. Specifically, our focus lies on studying the high-frequency trading data of the Bitcoin cryptocurrency spot on the LOB, an important component managed by the centralized exchanges that offers massive large-scale data. The LOB serves as a repository of bid and ask orders, and its detailed structure allows for a granular analysis of order book dynamics.

This approach is inspired from the principles of statistical mechanics and thermodynamics. The thermodynamics in the LOB is based on mathematical and conceptual parallels between physical complex systems and financial markets, providing an innovative perspective on market liquidity and volatility. Our methodology involves developing microscopic model on the orders in the LOB, defining the Active Depth of the LOB, quantifying the kinetic and potential energies, and interpreting Market Temperature and Market Entropy. The primary contributions of this study are two-fold.

*Thermodynamics of financial markets.* Our first contribution is the novel application of thermodynamic concepts on financial markets, particularly within the LOB. By treating the LOB as a thermodynamic system, this study interprets and quantifies the market dynamics through Market Temperature and Market Entropy. This provides inspirations for price discovery in market microstructure models, algorithmic trading strategies, and predictive modeling in financial markets. Our empirical findings not only broaden the theoretical framework of econophysics but also demonstrate the utility of physics principles in understanding the complex systems in finance.

*Understanding of market microstructure.* Second, our study contributes to an innovative understanding of order book dynamics and market participant behavior. This study discusses the implication of Market Temperature on conventional liquidity measures, including Active Quote Volume, bid–ask spread and match volume, and compares Market Entropy to Local Volatility, a proprietary method estimating the instantaneous volatility of price dynamics in high-frequency trading markets. The thermodynamics of the LOB reveals the way these liquidity and volatility measures evolve, which is beyond the ability of the time series of the LOB snapshot and price data that derives the liquidity and volatility measures.

The structure of this work is as follows. Section 2 outlines the evolution of econophysics, highlighting previous attempts to conceptualize financial markets using principles of physics, and introduces market measures and order book analysis. In Section 3, we detail the Level 3 order book datasets of event and snapshot data. Section 4 introduces the definitions of Active Depth, kinetic and potential energy within the LOB, forming the concepts of Market Temperature and Market Entropy in Section 5. Section 6 empirically evaluates these measures against indicators of market liquidity and volatility. Section 7 provides the theoretical implications, relation to literature, limitations and future directions of this study. The conclusions are drawn in Section 8.

## 2. Literature Review

The field of econophysics, integrating physics principles into financial economics, represents an interdisciplinary evolution, applying physical concepts to comprehend the statistical properties of financial markets. Borland’s exploration of the financial time-series and price dynamics models using physics to investigate the collective phenomena provided valuable insights in this area [1]. Loffredo’s review highlighted the significance of statistical physics in quantitative finance [2]. Potters and Bouchaud’s study on the statistical properties of order books revealed a logarithmic price impact function and proposed a model for order book dynamics, which underscored the lasting impact of trades and the importance of order flow in markets [3].

Earlier research in econophysics drew parallels between financial price fluctuation distributions and physical particle behaviors. Bouchaud and Cont’s work on stock market fluctuations applied Langevin dynamics to establish a statistical physics framework for flash crash analysis on market liquidity and volatility [4]. Ausloos’s analogy between the Boltzmann equation in physics and the Efficient Market Hypothesis (EMH) offered valuable insights [5]. Challet and Stinchcombe pioneered a particle model on the LOB [6,7], which investigated both static and dynamic properties of the LOB by treating orders as massive particles and price as position.

Naturally, the concept of temperature in thermodynamics was applied to characterize market behavior or volatility. Dragulescu and Victor argued that in a closed economic system where money is conserved, the equilibrium distribution of money aligned with the exponential Boltzmann–Gibbs law [8]. Kleinert and Chen demonstrated that S&P 500 and NASDAQ 100 indices followed Boltzmann statistics [9]; they used Market Temperature variations to analyze the US stock flash crash in 2000. Das discussed the intersection of financial theory, statistics, and thermodynamics [10], which validated the potential of leveraging such interdisciplinary approaches. Zambrano et al. applied the thermodynamics model based on the maximum entropy principle to study the financial problem of firms’ growth [11].

There are many studies focusing on the economic finding and market participant behavior in the LOB. Lee and Ready firstly studied the trading behavior of market participants by analyzing the order activities in the LOB [12]. Similar analysis of trading activities can be found in recent studies [13,14]. Cartea et al. investigated the imbalance between the bid and ask volumes [15] whereas Mendonca produced 1 min LOB snapshots from Level 2 order book data to detect spoofing activities [16]. Jiang et al. studied the order flow data on the dynamics of volatility, trading volume, and average trade size to analyze the trader’s behavior [17]. Zhang et al. studied the order imbalance to identify market participants and predict stock returns [18,19]. Yin and Wong engineered a deep convolutional neural network model to seize the information from the LOB data at a tick frequency of 0.5 s [20]. After exploiting such information, the performance of their trading algorithm proved that the LOB data contained rich information for liquidity, price prediction and trading signal generation.

The liquidity and volatility are the most valuable information that can be extracted from the LOB data. Holden et al. empirically examined different measures of market liquidity. They synthesized the liquidity by the quantity, cost and time dimensions [21]. In a different way, Aitken and Comerton-Forde categorized liquidity measures into trade based and order based by empirically evaluating their correlations and implications [22]. The value of market volatility cannot be underestimated given its substantial economic implications, as the fundamental objective of asset pricing demanded comprehension of the patterns of the volatility for expected return [23]. Zhang et al. empirically analyzed the issues of estimating integrated volatility in the presence of market microstructure noise in high-frequency data [24]. Hansen and Lunde examined the impact of market microstructure noise on volatility estimation and discussed various methods to mitigate its effects [25].

There is a growing interest into the order flow, price formation, and market microstructure to study the order book and market dynamics. Cont et al. firstly built a description of the order book dynamics microscopically [26,27]. With trailing of the limit order arrivals, cancellations and executions, their work enabled easier empirical analysis. Horst and Paulsen applied a similar method but studied the whole LOB instead of merely the top of the book (best bid and best ask quotes) [28]. Their work discussed a different scaling regime to approximate key order book statistics.

However, a critical research gap exists in the field of econophysics, particularly in the microscopic modeling of order activities within the LOB. Prior literature has primarily concentrated on the statistical properties of order book dynamics, often without investigating the individual order activities. Our study seeks to bridge this gap by developing a microscopic model on the orders within the LOB and derive the thermodynamic measures of Market Temperature and Market Entropy. This constructs a granular and deep understanding of LOB dynamics, which has been relatively unexplored in the context of econophysics.

Yura et al. studied the LOB by drawing a parallel between financial market dynamics and Brownian motion [29,30]. They modeled the bid–ask spread and the surrounding depths as a colloidal financial Brownian particle. Based on their work, Kanazawa et al. employed kinetic theories and derived the Boltzmann equation for financial Brownian motion [31,32]. Li et al. proposed a physics-based model conceptualizing orders as particles to derive the kinetic energy and momentum of the LOB [33,34]. This model aimed to describe the state of the system and was used to understand market participant behaviors, forecast volatility and price movements. Their empirical evaluations demonstrated that this model outperformed traditional methods and machine learning algorithms in predicting volatility and price movements.

## 3. Data

For this empirical study, we process and analyze Level 3 order book event data and LOB snapshot data of Bitcoin cryptocurrency spot. The order book event dataset contains comprehensive records of incoming order activities of submission, cancellation, update and match. An event-based record with timestamp is created each time one of these order activities takes place. The snapshot dataset is constituted by the quoted prices and aggregated quote volume of existing bid and ask orders. The snapshot data reflect the detailed overview of the LOB and are recorded every time the LOB is updated by the centralized exchanges.

Our data are considered to be high-frequency trading data (in microseconds) sourced from the Coinbase exchange, accessed via Websocket feeds. Due to the nature of thermodynamic modeling, this study requires large-scale event data to model them with physical particle dynamics. As a result, we study the Bitcoin/USD pair, as it has the largest trading volume among existing cryptocurrencies. There are only limited exchanges that provide the Level 3 order book event data, and Coinbase is the most popular exchange; hence, it offers the largest amount of available data demanded by this study.

The high-frequency nature of the dataset resulted in a significantly large amount of data points, even for a relatively short timeframe. Specifically, we collected two datasets whose amounts of datapoints are as listed in Table 1, and the statistical analysis of price time series for each subset are shown in Table 2.

In our recorded dataset, the price granularity is fixed at USD 0.01, with minimum trading volumes of BTC 0.00001 for the BTC/USD pair. Timestamps are recorded to microsecond precision for the event-driven records of order activities. In our previous study, we integrated the event data with a sampling frequency of Δt=0.1 s and 1 s for snapshot data. However, the nature of thermodynamic methodology demands a granular level of individual order activities. As a result, this study will not sample the datasets in favor of preserving the event-driven nature of the data, thereby capturing information at the most granular level. This adjustment ensures fidelity to the thermodynamic framework and the comprehensive analysis of microscopic order book dynamics.

## 4. Theoretical Framework

In this section, we investigate the concept of applying thermodynamic principles to model the LOB. We explore the analogy between the physical concepts of kinetic energy and potential energy with giving their definitions and theoretical underpinnings within the context of the LOB. Building on the concept of kinetic energy from our previous work, we now define the potential energy, in preparation for adapting Market Temperature and Market Entropy concepts in Section 5. This approach aims to provide a more comprehensive understanding of the LOB dynamics by interpreting its behavior through developing microscopic model on the orders in the LOB.

### 4.1. Basic Concepts

In our framework, the orders in the LOB are conceptualized as particles with motion on the one-dimensional LOB. The order size corresponds to the particle mass, while the distance it moves is the price displacement on the LOB.

We define the velocity of orders as the absolute distance between its quoted price and bound of Active Area divided by the sampling time interval. We treat the time difference as unit time when we calculate the velocity of each order. As a result, for an order that is quoted at price $p^{*}\left(t\right)$, its velocity is given by its quoted price, best bid price, best ask price, and Active Depth α given in Equation (7):

(1) $$v\left(t\right)=p^{*}\left(t\right)-\left(b_{M}\left(t\right)-\alpha \right)$$

for bid orders, where $b_{M}$ is the best bid price, and

(2) $$v\left(t\right)=\left(a_{M}\left(t\right)+\alpha \right)-p^{*}\left(t\right)$$

for ask orders, where $a_{M}$ is the best ask price.

The depth of the order book specifies the location of a quoted price in the LOB. At time *t*, the depth of a specific limit order quoted at price $p^{*}$ is denoted by $\gamma (p^{*},t)$ and defined by

(3) $$\gamma \left(p^{*},t\right)=\left[b_{M}\left(t\right)-p^{*}\right]$$

for the bid side, where the quoted price of bid orders are lower than the best bid price $b_{M}$. And

(4) $$\gamma \left(p^{*},t\right)=\left[p^{*}-a_{M}\left(t\right)\right]$$

for the ask side, where the quoted price of ask orders is higher than the best ask price $a_{M}$. We denote the total number of distinct limit orders quoted at a specific depth γ at time *t* in the LOB by $n_{\gamma }\left(t\right)$.

We define the bid volume as the total volume of quoted orders inside depth γ at time *t* as:

(5) $$V_{\mathrm{bid}}\left(\gamma \right)=\sum_{\gamma^{*}=b_{M}-\gamma }^{0}\sum_{i=0}^{n_{\gamma }\left(t\right)}s_{i}$$

for the bid side of the LOB, where $s_{i}$ is the order size of the $i^{th}$ limit order quoted at this depth.

Similarly, we define the ask volume as

(6) $$V_{\mathrm{ask}}\left(\gamma \right)=\sum_{\gamma^{*}=0}^{a_{M}+\gamma }\sum_{j=0}^{n_{\gamma }\left(t\right)}s_{j}$$

for the ask side of the LOB.

After sampling at a frequency of one second on the snapshot datasets, we record the mid price *p* along with the bid volume $V_{\mathrm{bid}}$ and the ask volume $V_{\mathrm{ask}}$ as in Equations (5) and (6). We iterate on the depth γ through the LOB to calculate the change in these two variables and have Δp, $\Delta V_{\mathrm{bid}}\left(\gamma \right)$, and $\Delta V_{\mathrm{ask}}\left(\gamma \right)$.

The concept of Active Area with Active Depth α represents those depths around the bid–ask spread that have the most active order activities in the LOB. Within this specified range, there are the highest level of activities for order submissions, cancellations, updates and matches. In contrast, the limit orders outside of this range tend to remain relatively insensitive to the market price movements. And the terms ‘active bid price’ and ‘active ask price’ are defined as the best bid price minus α, and the best ask price plus α, separately. In our proceeding work, we proved that the Active Depth is 50 for Bitcoin/USD markets [34], using the method calculating the cross-correlation coefficients between the total volume of reacted orders (either canceled or submitted) and the price change for each depth in the LOB. This finding is validated in our study with the following distinct method.

**Figure 1:**
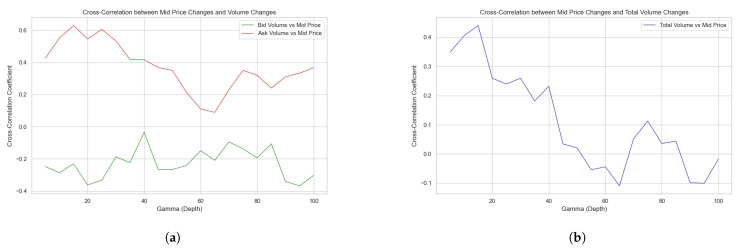
Cross-correlation functions between the price and the quote volume in varying LOB depths. (**a**) Price and the bid/ask side quote volume. (**b**) Price and the total quote volume.

This study carries out analysis on the cross-correlation function $\mathrm{Corr}[\Delta p,\Delta V_{\mathrm{bid}}\left(\gamma \right)]$ and $\mathrm{Corr}[\Delta p,\Delta V_{\mathrm{ask}}\left(\gamma \right)]$, which serves to unravel the interplay between the mid price movement and the corresponding response of quoted orders inside depth γ. These cross-correlation function are demonstrated in Figure 1a.

Figure 1a presents a graphical analysis of the cross-correlation coefficients between mid-price changes and changes in both bid volume $\Delta V_{\mathrm{bid}}$ and ask volume $\Delta V_{\mathrm{ask}}$ across various depths γ in the LOB. There is a positive cross-correlation coefficient between the ask volume changes and mid-price movements, especially at lower depths. This suggests that as the price move up, the volume of ask orders (sell side) closely follows, implying a reactive adjustment by sellers in the LOB. Similarly, the negative cross-correlation coefficient for bid volume changes indicates that as the price move down, the bid orders also react accordingly. As both cross-correlation coefficients approach zero, it represents that in the deeper depths far away from the bid–ask spread, the bid and ask orders stop reacting to the price movements. Additionally, we compute $\mathrm{Corr}[\Delta p,\Delta \left(V_{\mathrm{bid}}\left(\gamma \right)+V_{\mathrm{ask}}\left(\gamma \right)\right)]$ as in Figure 1b, which shows a more apparent trend.

Figure 1b examines the cross-correlation between mid-price changes and the total volume changes within the LOB, consolidating both bid and ask volumes. The cross-correlation starts positive at lower depths but shows a decline as the depth increases, eventually fluctuating around zero. This trend reversal could imply that the aggregated quote volume across both sides of the book is less predictive of mid-price movements in deeper depths of the LOB.

Empirically, we can define Active Depth α by:

(7) $$\alpha =\underset{\gamma }{\arg}\;\left\{\mathrm{Corr}[\Delta p,\Delta \left(V_{\mathrm{bid}}\left(\gamma \right)+V_{\mathrm{ask}}\left(\gamma \right)\right)]=0\right\}$$

and Active Quote Volume (AQV) as:

(8) $$\mathrm{AQV}=\sum_{\gamma \in \alpha }V_{\mathrm{bid}}\left(\gamma \right)+V_{\mathrm{ask}}\left(\gamma \right))$$

In Figure 1b, it is apparent that α is approximately 50 when $\mathrm{Corr}[\Delta p,\Delta \left(V_{\mathrm{bid}}\left(\gamma \right)+V_{\mathrm{ask}}\left(\gamma \right)\right)]$ reaches 0.

The findings from these results reveal the heterogeneous nature of order book dynamics that varies across the depth in the LOB in response to price movements. The initial positive correlation at lower depths aligns with the common view that the depths around bid–ask spread are occupied with the most order activities in the LOB. At the depths far from the mid-price, the correlation diminishes, reflecting the decreased sensitivity and less liquidity provided by market participants. The analysis validates the Active Depth of 50 for the Bitcoin/USD market. The Active Depth could serve as an important parameter for market participants, especially market makers, in optimizing their algorithms for order quotes and cancellations around this depth.

### 4.2. Kinetic Energy

In this subsection, we discuss how kinetic energy, traditionally associated with the dynamics of physical particles by $\frac{1}{2}\cdot m\cdot v^{2}$, can be defined and analogized to the dynamic behavior of the LOB, particularly the rapid submission and cancellation of orders in the LOB. Its calculation is derived from the event data.

For each order either submitted or canceled from the Active Area, we define its kinetic energy at time *t* as $E=\frac{1}{2}\cdot s\cdot v^{2}$, where *s* is the order size and *v* is the velocity. As a result, we may calculate the total changed in the kinetic energy (*KE*) of the LOB between time $t_{1}$ and $t_{2}$ with:

(9) $$\Delta KE\left(t_{1},t_{2}\right)=\int_{t=t_{1}}^{t_{2}}\sum_{\gamma =b_{M}-\alpha }^{a_{M}+\alpha }\left(\sum_{i=0}^{N_{\gamma }\left(t\right)}\frac{1}{2}s_{i}\cdot {v_{i}}^{2}+\sum_{j=0}^{N_{\gamma }^{\prime }\left(t\right)}\frac{1}{2}s_{j}^{\prime }\cdot {v_{j}^{\prime }}^{2}\right)$$

where $s_{i}$ and $v_{i}$ are the order size and velocity of the *i*th submitted limit order quoted at this depth, and $s_{j}^{\prime }$ and $v_{j}^{\prime }$ are for the *j*th canceled limit order quoted at this depth.

**Figure 2:**
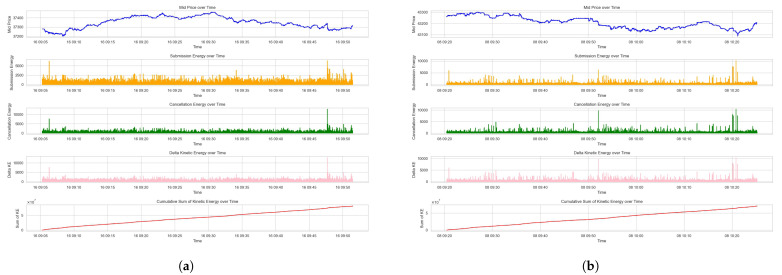
Kinetic energy measures of the LOB. (**a**) Kinetic energy measures for Dataset 1. (**b**) Kinetic energy measures for Dataset 2.

The resulted plots on the Dataset 1 and Dataset 2 are shown in Figure 2a,b, which include the mid price, the part of kinetic energy that is caused by order submission and cancellation, and the kinetic energy, as well as the cumulative sum of kinetic energy.

Figure 2 showcases the interplay between the mid-price movements and associated kinetic energies over the given time period. The submission energy, representing the energy from new limit orders, shows sporadic peaks that correspond to increased trading intent. These peaks may signify the arrival of new information or a collective response to market events, prompting traders to realize their quoting interests. The cancellation energy subplots, on the other hand, illustrate the energy removed from the market due to order withdrawals. Sharp increases in cancellation energy could imply a shift in market sentiment or a reaction to the fulfillment of short-term trading strategies.

The overall kinetic energy graph aggregates the energies from submissions and cancellations. It provides a composite view of market activity intensity, where higher values denote greater market agitation. The cumulative sum of kinetic energy offers a sense of the market’s directional momentum over time. The gradual upward trend indicates a market accumulating potential energy.

The kinetic energy (*KE*) measure captures the dynamics of order activities. It reflects the movement and quoted size of orders within the LOB, capturing their immediate and active impact on the market microstructure.

### 4.3. Potential Energy

As for the potential energy in the LOB, it is computed with the snapshot data based on the Active Area, analogous to the potential energy in the physics of m·g·h. For the bid side, the potential energy ($PE_{\mathrm{bid}}$) at time *t* is the summation of the product of order size, gravity constant *g* and its distance to the active bid price for each order inside the Active Area. It is expressed as:

(10) $$PE_{\mathrm{bid}}\left(t\right)=\sum_{\gamma =b_{M}-\alpha }^{0}\sum_{k=0}^{N_{\gamma }\left(t\right)}s_{k}\cdot g\cdot \left(p_{k}^{*}\left(t\right)-\left(b_{M}\left(t\right)-\alpha \right)\right).$$

Similarly, for the ask side, the potential energy ($PE_{\mathrm{ask}}$) is given by:

(11) $$PE_{\mathrm{ask}}\left(t\right)=\sum_{\gamma =0}^{a_{M}+\alpha }\sum_{k=0}^{N_{\gamma }\left(t\right)}s_{k}\cdot g\cdot \left(\left(a_{M}\left(t\right)+\alpha \right)-p_{k}^{*}\left(t\right)\right).$$

For simplifying the computations in the later section, we express them as:

(12) $$PE_{\mathrm{bid}}\left(t\right)=f_{\mathrm{bid}}\left(t\right)\cdot g$$

and

(13) $$PE_{\mathrm{ask}}\left(t\right)=f_{\mathrm{ask}}\left(t\right)\cdot g.$$

In general, the potential energy (PE) is linked to the depth of the LOB. This measure reflects the position (distance to the bid–ask spread) and quoted size of the existing limit orders within the Active Area, which indicate the potential impact to the bid–ask spread and the shape of the LOB.

In order to compute the gravity constant *g* for the LOB, we select a short time interval (2023-11-16 09:19:30–09:20:30) of the data during which the price and the LOB stayed relatively static and stable. We define the net potential energy as

(14) $$\mathrm{net}\;PE\left(t\right)=PE_{\mathrm{bid}}\left(t\right)-PE_{\mathrm{ask}}\left(t\right)$$

which represent the imbalance of the order book in the bid side and ask side. As the price and order book dynamics in the LOB are impacted by, and only by, the existing limit orders and incoming orders, reasonably we assume that the sum of net potential energy and sum of kinetic energy during this time interval maintains relative equilibrium. Mathematically,

(15) $$\Delta KE\left(t_{1},t_{2}\right)=\mathrm{net}\;PE\left(t_{2}\right)-\mathrm{net}\;PE\left(t_{1}\right)=\left[PE_{\mathrm{bid}}\left(t_{2}\right)-PE_{\mathrm{ask}}\left(t_{2}\right)\right]-\left[PE_{\mathrm{bid}}\left(t_{1}\right)-PE_{\mathrm{ask}}\left(t_{1}\right)\right]$$

As a result, substituting with Equations (9), (12) and (13), we can compute the gravity constant *g* by

(16) $$g=\frac{\int_{t=t_{1}}^{t_{2}}\sum_{\gamma =b_{M}-\alpha }^{a_{M}+\alpha }\left(\sum_{i=0}^{N_{\gamma }\left(t\right)}\frac{1}{2}s_{i}\cdot {v_{i}}^{2}+\sum_{j=0}^{N_{\gamma }^{\prime }\left(t\right)}\frac{1}{2}s_{j}^{\prime }\cdot {v_{j}^{\prime }}^{2}\right)}{\left[f_{\mathrm{bid}}\left(t_{2}\right)-f_{\mathrm{ask}}\left(t_{2}\right)\right]-\left[f_{\mathrm{bid}}\left(t_{1}\right)-f_{\mathrm{ask}}\left(t_{1}\right)\right]}$$

According to the data where $t_{2}$ = 09:20:30 and $t_{1}$ = 09:19:30, we calculate the result *g* = 0.2958149403, which can be used for calculating the accurate potential energy in the latter sections. The computed gravity constant *g* serves as a scaling factor for potential energy, enabling a standardized assessment of market dynamics.

**Figure 3:**
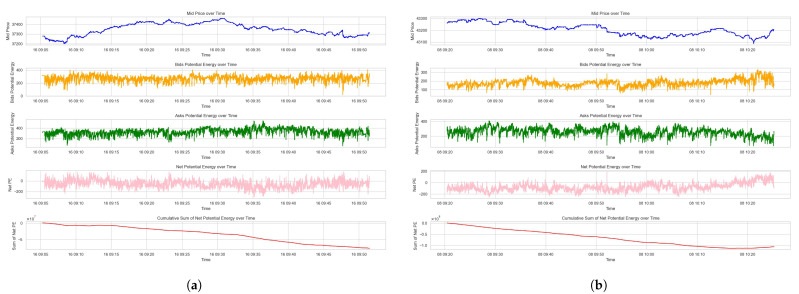
Potential energy measures of the LOB. (**a**) Potential energy measures for Dataset 1. (**b**) Potential energy measures for Dataset 2.

The results on the potential energy can be seen in Figure 3 showing the mid price, bid and ask side potential energy, net potential energy, and the cumulative sum of net potential energy for Dataset 1 and Dataset 2. In the figures, the second and third subplots demonstrate the potential energy for bid and ask sides over time, which fluctuate around a mean level, indicating the continuous adjustment of market participants’ quotes relative to price changes. The fourth subplots of the net potential energy reveal the temporal asymmetry in the market liquidity on the bid and ask sides in the LOB, which is impacted by the supply and demand on the underlying asset.

Note that the total potential energy is defined by

(17) $$\mathrm{total}\;PE\left(t\right)=PE_{\mathrm{bid}}\left(t\right)+PE_{\mathrm{ask}}\left(t\right)$$

## 5. Thermodynamics of the LOB

### 5.1. Market Temperature

In the realm of financial markets, we introduce the concept of Market Temperature as an indicator of the market’s overall energy state, derived from both kinetic and potential energies present in the LOB. The Market Temperature, adapted from thermodynamics, offers a novel lens to view LOB dynamics.

In physical systems, temperature quantifies the average kinetic energy of particles. By analogy, in financial markets, especially in the LOB, temperature is metaphorically akin to a measure of the market’s energy dynamics. This energy encompasses the actions and interactions within the market, analogous to the kinetic and potential energies in physical systems.

In thermodynamics, the change in a system’s temperature is primarily influenced by the change in the system’s internal energy, which includes all the energy contained within it. This contains the kinetic energy due to the motion of particles and potential energy resulting from forces within the system or between its components. The change in internal energy is due to heat transfer or work performed by the system. Mathematically, for a closed system, the first law of thermodynamics expresses this relationship as:

(18) ΔU=Q−W

where ΔU is the change in internal energy, *Q* is the heat added to the system, and *W* is the work done by the system [35,36].

The temperature of the LOB, denoted as *T*, is a composite measure that integrates both the kinetic energies of incoming orders, and the potential energies associated with existing limit orders.

To compute the temperature of the LOB, we neglect the other factors that influence the temperature except the internal energy so that we have ΔT=ΔU. For the internal energy, in the view of physics, the LOB does not do any work to the outside but only experiences the heat transfer through order submission and cancellation. As a result, ΔT=Q, and it is only influenced by the change in the kinetic energy (*KE*), as well as the potential energy (*PE*) on both the bid and ask sides. The formula is expressed as:

(19) $$\Delta T=\Delta {\mathrm{PE}}_{\mathrm{bid}}+\Delta {\mathrm{PE}}_{\mathrm{ask}}+\Delta \mathrm{KE}.$$

Here, ${\mathrm{PE}}_{\mathrm{bid}}$ and ${\mathrm{PE}}_{\mathrm{ask}}$ represent the potential energies on the bid and ask sides of the market, respectively, and KE denotes the kinetic energy of the orders.

**Figure 4:**
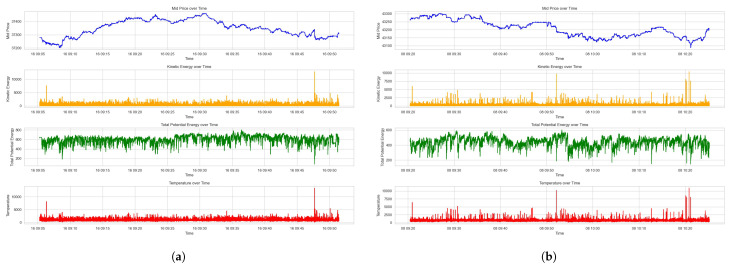
Market Temperature of the LOB. (**a**) Market Temperature for Dataset 1. (**b**) Market Temperature for Dataset 2.

The plots of mid price, kinetic energy, total potential energy, and temperature are demonstrated in Figure 4.

The second subplots in Figure 4 represent the kinetic energy in the LOB, likely quantifying the vigor of market participants’ actions, such as order placement, modification, or cancellation. Peaks in this subplot may correspond to bursts of trading activity, which could be driven by market events or periods of high volatility. The third subplot measures the total potential energy in the LOB, reflecting the accumulation of limit orders. Consistent levels of potential energy suggest a balanced market with stable liquidity, whereas significant variations imply an increase in the quoted orders on the LOB. Observing the changes in potential energy could be critical for understanding the market’s capacity to absorb large trades without significant impact on the price.

The final subplot is a novel representation of Market Temperature, a composite measure designed to capture the overall energy state of the market by combining kinetic and potential energies. An increase in Market Temperature could indicate increased market activity, possibly leading to greater price volatility and risk. Conversely, a lower or stable temperature might suggest a less active market, potentially offering a more stable environment for execution.

The synchrony of these bursts across kinetic energy, potential energy, and temperature plots suggests a significant and possibly unexpected market event. This is possibly a liquidity event, where the limit orders at current prices are suddenly taken up by market orders, possibly leading to jumps in best bid or best ask quotes and the gap in bid–ask spread.

### 5.2. Market Entropy

In this section, the implementation of the concept of Market Entropy is proposed.

Entropy (*S*) is a fundamental concept in thermodynamics that measures the disorder or randomness in a system. It is important in understanding the behavior of physical systems. The second law of thermodynamics states that in an isolated system, the entropy tends to increase over time, reaching a maximum at the equilibrium state. This law reflects the natural tendency of systems to evolve towards the states with higher disorder and randomness.

For reversible processes, the change in entropy (ΔS) is defined as the heat transfer (ΔQ) to or from the system, divided by the temperature (*T*) at which this transfer occurs [37]. Mathematically, this is expressed as:

(20) $$\Delta S=\frac{\Delta Q}{T}=\frac{\Delta T}{T},$$

where ΔT is the increment of temperature to the system caused by each order activity and *T* expresses its aggregation. From the perspective of statistical mechanics, the measure of entropy provides a link between the microscopic properties of particles and the macroscopic observable properties of the system.

In the LOB context, the ‘heat transfer’ to or from the system can be interpreted as the ‘energy transfer’ within the LOB that is due to the order activities. The order submission can be viewed as the heat transferred into the LOB, and the order cancellation as the heat transferred outside from the LOB. These dynamics can be computed with the changes in kinetic and potential energy due to order activities such as placements, modifications, or cancellations.

In our model, we interpret changes in kinetic and potential energies in the LOB as ‘energy transfer’ within the market. As a result, the aggregated entropy for from time $t_{1}$ to $t_{2}$ is defined as:

(21) $$S=\sum_{t=t_{1}}^{t_{2}}\Delta S=\sum_{t=t_{1}}^{t_{2}}\frac{\Delta Q}{T}=\sum_{t=t_{1}}^{t_{2}}\frac{\Delta T}{T},$$

where ΔQ represents the change in kinetic and potential energies in the LOB, and *T* is the aggregated Market Temperature. Note that ΔT represents the instantaneous changes in the Market Temperature that are caused by individual event-based order activities. It implies the immediate impact of the LOB dynamics extracted from the event-based data. The summation is performed over a specified time interval $\left(t_{1},t_{2}\right)$.

**Figure 5:**
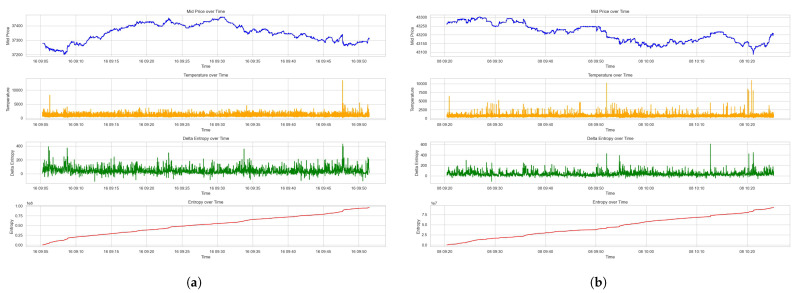
Market Entropy of the LOB sampled by 1-second frequency. (**a**) Market Entropy for Dataset 1. (**b**) Market Entropy for Dataset 2.

We derive the aggregated results in Figure 5 after sampling Dataset 1 and Dataset 2 at a frequency of 1 s. As shown in the plots, sampling at a 1 s frequency introduces a time-averaging effect, which could smooth out some of the rapid fluctuations seen in the event-based data. This sampling frequency might filter out the noise of less significant events, providing a clearer signal for the overall entropy dynamics of LOB.

The second subplots in Figure 5 display Market Temperature, an analogue to thermodynamic temperature, reflecting the intensity of market activity and energy states within the LOB. The third subplots displays the delta entropy, representing the instantaneous change in market disorder. Peaks here likely correspond to significant market events that induce rapid shifts in the market microstructure. The final subplots illustrate the cumulative Market Entropy. An upward trend signifies an increase in disorder, implying that the market is always moving away from equilibrium.

## 6. Empirical Validation

### 6.1. Market Liquidity

In empirical finance, liquidity measures are important for investigating the market microstructure and LOB dynamics. Holden et al. categorized the liquidity measures by the cost, quantity and time dimensions [21]. In this study, we show the implication of the Market Temperature on market liquidity by evaluating three liquidity measures of Active Quote Volume, bid–ask spread, and match volume.

The Active Quote Volume (AQV), defined in Equation (8), is a quantity dimension measure aggregating the quoted volume of bid and ask orders within a specified depth in the LOB, specifically, inside the Active Depth discussed in Section 4.1, reflecting the market’s capacity to absorb large orders without significant price impact.

The bid–ask spread, representative of the cost dimension, quantifies the immediate trading cost for liquidity takers engaging in round-trip trades, where they buy at the best ask price and sell at the best bid price. Irvine et al. validated the Cost of Round Trip (CRT) trade as a comprehensive liquidity measure [38]. This spread is key in estimating the efficiency and competitiveness of a market.

Lastly, the match volume, in the time dimension, aggregates the volume of matched orders over a given time interval, offering insights into the market’s temporal liquidity flow and the degree of trading activities. Each of these measures provides a unique perspective on market liquidity, contributing to a more comprehensive understanding of market participants’ behavior and efficiency.

As for the category of Aitken and Comerton-Forde [22], the Active Quote Volume and bid–ask spread are order-based liquidity measures, while the match volume is trade based.

**Figure 6:**
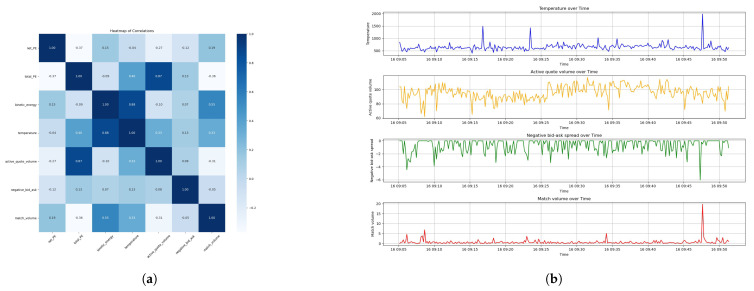
Analysis of thermodynamic and liquidity measures of the LOB for Dataset 1. (**a**) Heat map of the different measures. (**b**) Plots of Market Temperature and liquidity measures.

**Figure 7:**
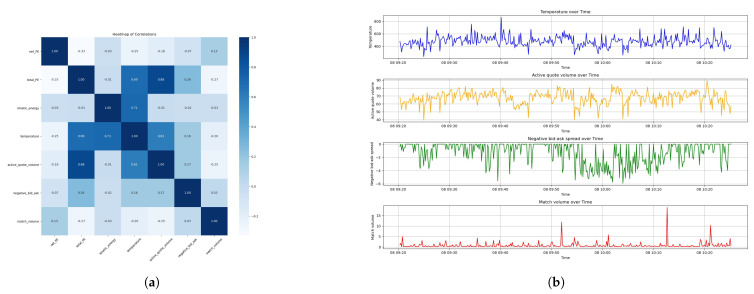
Analysis of thermodynamic and liquidity measures of the LOB for Dataset 2. (**a**) Heat map of the different measures. (**b**) Plots of Market Temperature and liquidity measures.

Note that a higher Active Quote Volume or match volume indicates better liquidity, while it is the opposite for the bid–ask spread. The bid–ask spread is a positive number since it is conventionally computed by the best ask price minus the best bid price. As a result, we study the negative of the bid–ask spread, or say ‘ask–bid’ spread, in order to positive-proportionally align with the Market Temperature. We carry out Pearson correlation analysis on the physics measures and the liquidity measures after resampling the data with a frequency of 10 s. The heatmap and the plots of thermodynamic measures and liquidity measures are shown in Figure 6a,b for Dataset 1, and Figure 7 for Dataset 2.

The comprehensive analysis presented in Figure 6 for Dataset 1 reveals intriguing correlations that are important in understanding market dynamics from a microstructural perspective. The correlation coefficient between Market Temperature and Active Quote Volume stands at a moderate 0.33, indicating a positive but not dominant relationship. This suggests that as the Market Temperature rises, we can expect a proportionate increase in the quoted volume, implying an elevated capacity for the market to absorb order flows without substantial price adjustments. It is similar for a match volume with a coefficient of 0.33, suggesting that temperatures could be a indicator of the temporal trading activity.

Concurrently, the temperature’s correlation with the negative bid–ask spread is 0.13, underscoring a slight inverse relationship between the Market Temperature and the immediate trading cost by liquidity takers. Although the correlation is not strong, it still implies that temperatures could be associated with marginally reduced trading costs, showing better market liquidity.

Meanwhile, Dataset 2, as demonstrated in Figure 7a,b, showcases a stronger positive correlation of 0.61 between the temperature and Active Quote Volume. This robust relationship points the effect of Market Temperature on the market’s capacity to handle larger orders, which is proof of the market’s liquidity resilience.

In Dataset 2, the negative bid–ask spread correlation with temperature at 0.16 is slightly higher than in Dataset 1. However, the correlation between the temperature and match volume turns negative, at −0.2, for Dataset 2. This result that is opposite to Dataset 1 indicates that the relationship between the temperature and match volume could be unstable and changes at different market states. The mechanism and reason behind is due to be discovered in the future studies.

The data analysis on Market Temperature allows a more granular understanding of the thermodynamics of LOB. It implies that the Market Temperature is related to liquidity measures, each with its distinct behavioral pattern.

### 6.2. Market Volatility

Different market volatility measures, when applied to the same time series data of price, can yield varied outcomes. Cont et al. underscored this phenomenon, noting that these measures demonstrate distinct intra-day behavior patterns across various assets [39]. In the context of high-frequency trading, the volatility measure’s effectiveness is often disturbed by the ‘market microstructure noise’, a phenomenon emerging from the irregular and discrete price jumps across the order book data ticks. Zhang et al. [24] and Hansen and Lunde [25] validated the impact of ‘market microstructure noise’ on volatility estimation.

The traditional calculation of volatility involves computing the standard deviation of returns, which is based on the mean return. However, when the asset price exhibits a trending behavior, the mean return could be significantly different from zero, and changing the length of the time window used for the calculation could result in artificially high volatility values. This is because more returns would be further away from the mean, leading to a larger sum of squared deviations.

When it comes to computing market volatility, a pivotal measure in financial econometrics, the literature has conventionally relied on the standard deviation of price data. Meanwhile, if the asset prices are characterized by trending behaviors, the mean return of price could deviate substantially from zero. Extending the temporal time window for computation could result in significant deviations from the mean and lead to unnecessarily high values, while the price data themselves do not change. These problems are even worse for the high-frequency trading data. As a result, the traditional volatility measures conventionally applied on mid/low-frequency price data do not apply to the order book data used in this study.

To solve this problem, the investigation introduces an alternative volatility measure—the Local Volatility—as the benchmark. We adopt a proprietary method to calculate the instantaneous volatility, which was described in our previous work [34].

This volatility measure deals with the tick data, where the price changes in a microsecond frequency between successive event time (ticks), and computes the standard deviation of the increments rather than the returns between the prices at consecutive ticks. This measure focuses on the changes of price from a tick to the next, instead of the overall trend.

The Local Volatility $\sigma_{LV}$ of price series $P_{t}$ is defined as:

(22) $$\sigma_{LV}=\sqrt{\frac{\sum_{t=1}^{T}{\left(P_{t}-P_{t-1}\right)}^{2}}{T}}$$

where $P_{t}$ stands for the price at time *t*, among the set of price observations *T*.

This mathematical formula estimates the variance of successive price differences ($P_{t}-P_{t-1}{)}^{2}$, aggregates them, and subsequently normalizes this sum by the set of observations to yield the mean squared difference, and finally takes the square root to obtain the standard deviation of price changes. The extraction of the square root gives the standard deviation of these price increments. The Local Volatility measure offers a practical estimation of the instantaneous volatility of price time-series data, particularly with a high frequency. We derive and estimate such Local Volatility to ensure Market Entropy is compared with the accurate and fair volatility in high-frequency trading markets.

**Figure 8:**
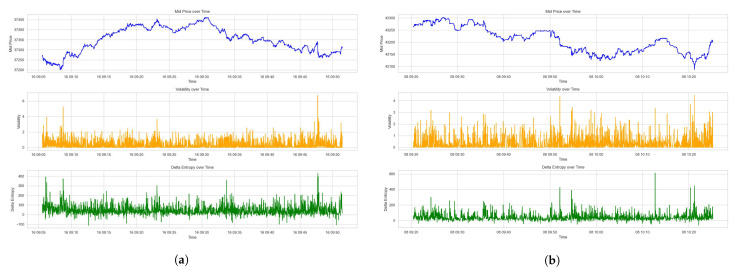
Price, Local Volatility and Delta Entropy of the LOB. (**a**) Plots for Dataset 1. (**b**) Plots for Dataset 2.

The plots on the price movements, the corresponding Local Volatility $\sigma_{LV}$ with sampling buffer *T* of 10 s (Equation (22)) and Delta Entropy ΔS (Equation (20)) are depicted in Figure 8 for Dataset 1 and Dataset 2.

In the estimation of the relationship between Local Volatility $\sigma_{LV}$ and Delta Entropy ΔS, we conduct a series of the following regressions independently for Dataset 1 and Dataset 2:

(23) $$\Delta \sigma_{LV}=\alpha_{1}+\beta_{1}\Delta S+\epsilon .$$

for Dataset 1, and

(24) $$\Delta \sigma_{LV}=\alpha_{2}+\beta_{2}\Delta S+\epsilon .$$

for Dataset 2.

**Figure 9:**
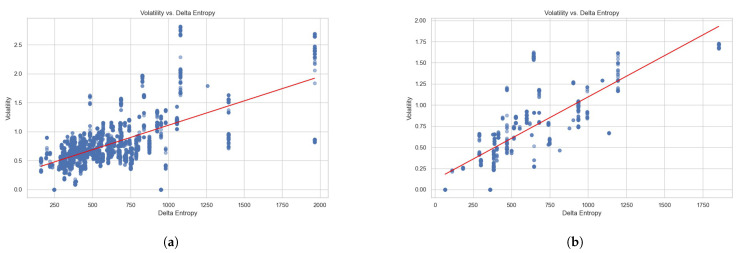
Regression analysis of Local Volatility and Delta Entropy. (**a**) Regression analysis for Dataset 1. (**b**) Regression analysis for Dataset 2.

Each dataset of the order book data is sampled by 10-second frequency; the sampled data comprise 27,716 and 38,880 data points for Dataset 1 and Dataset 2, separately. The performance results for these regression analyses are shown in Figure 9. The Ordinary Least Squares regression (OLS) results are detailed in Table 3 and the fitted parameters of regressions are listed in Table 4.

The regression models present encouraging insights, empirically evidenced by the substantive correlation coefficients of 0.644 and 0.752 for Datasets 1 and 2, respectively. These coefficients reflect the degree to which the Delta Entropy (ΔS) explains the Local Volatility ($\sigma_{LV}$), with the models accounting for 41.4% and 56.6% of the variance in volatility for the respective datasets. The statistical significance of these findings is validated by the robust F-statistics and the negligible p-values, underscoring the predictive power of Delta Entropy (ΔS) in explaining the observed Local Volatility ($\sigma_{LV}$).

The skewness and kurtosis, as captured by the Omnibus and Jarque–Bera statistics, implies a distribution of the Local Volatility ($\sigma_{LV}$) that deviates from the Gaussian ideal, particularly in Dataset 1. This skew towards higher volatility events hints at the presence of outlier dynamics within the market microstructure, possibly indicative of large-scale market events or the behaviors of informed traders.

In summation, this study not only empirically validates the Local Volatility ($\sigma_{LV}$) measure as an accurate tool for describing high-frequency trading data but also proves Delta Entropy (ΔS) as a significant indicator of volatility. These insights provide implications on understanding the market microstructure and LOB dynamics.

## 7. Discussion

The exploration of Market Temperature and Market Entropy within the LOB presents a novel approach in econophysics, which studies the intricate dynamics of order book through a thermodynamic lens. The application of these concepts to high-frequency trading data, particularly in the context of the Bitcoin cryptocurrency spot, offers an innovative perspective on market behavior and its underlying dynamics.

The concept of Market Temperature, as derived from the kinetic and potential energies of the LOB, provides a comprehensive measure of the market’s overall energy state. This measure captures the intensity of market activities, analogous to physical temperature in thermodynamics. It reflects the market’s activity and volatility, offering insights into the liquidity. The observed relationship between Market Temperature and order book dynamics highlights the potential of this measure in anticipating market behavior, providing a useful tool for market participants to gauge the market’s state. Furthermore, the introduction of Market Entropy, which quantifies the disorder or randomness in the LOB, unveils the degree of complexity and uncertainty in market dynamics. The increase in entropy might signal upcoming market fluctuations or shifts, thereby serving as an indicator of market stability.

The integration of these thermodynamic measures into market analysis presents an opportunity to enhance traditional financial models, which often overlook the complex and nonlinear nature of market dynamics. By applying the concepts from physics, this study contributes to a more comprehensive understanding of the LOB, providing a framework for more sophisticated analyses and forecasts.

### 7.1. Theoretical Implications

The application of Market Temperature and Market Entropy within the LOB offers a groundbreaking approach in econophysics, merging the intricate dynamics of financial markets with thermodynamic principles. Particularly in the realm of high-frequency trading data of cryptocurrencies like Bitcoin, these concepts provide an innovative lens to examine market behavior and its complex underpinnings.

As proposed, the temperature of the LOB is a function of its kinetic and potential energies. It reflects the intensity and activity level of order book dynamics, analogous to the physical concept of temperature. It could indicate the market liquidity and serve multiple functions:

- Analyze market conditions: Higher temperatures indicate potentially increased LOB capacity and order activities, while lower temperatures suggest a relatively less active and dynamic market state.
- Estimate market liquidity: Temperature can provide insights into the liquidity of the market. High temperatures often correlate with higher liquidity resulted from the market microstructure of the LOB.
- Algorithmic trading strategies: Understanding the temperature of the market can help market participants, informing and optimizing the liquidity trading strategies according to the Market Temperature levels.

Incorporating the concept of temperature into the analysis of the LOB offers a unique perspective on order book dynamics and view of market behavior. By understanding the temperature of the LOB, market participants can gain insights into the market microstructure and enhance their decision-making processes.

The proposed quantification of entropy in the LOB offers a novel perspective on market behavior. By assessing the degree of disorder or randomness in the order activities, we gain insights into the underlying dynamics of financial markets. A higher increment of the entropy measure may indicate a market characterized by rapid and diverse order activities, reflecting a state of high uncertainty or volatility. Conversely, a lower increment of the entropy measure could indicate a more stable market.

The key implications of Market Entropy include:

- Market stability analysis: By tracking changes in entropy, we can identify regimes of market stability or instability. If sudden increases are observed in the entropy measure, it might signal upcoming market fluctuations or shifts in market state.
- Trade optimization: For high-frequency trading algorithms, especially of market makers, entropy can serve as a robust indicator and parameter of the instantaneous volatility, for optimizing bid and ask quotes and ensuring that they adapt to varying market volatility.

### 7.2. Relation to the Literature

This study’s theoretical framework is based on the foundational works in econophysics, where parallels are drawn between LOB and thermodynamic systems. The use of Market Temperature and Market Entropy aligns with the research of Yura et al. [29,30] and Kanazawa et al. [31,32], which integrate physical concepts to explain the order book dynamics. This study extends their work by quantifying LOB dynamics using thermodynamic measures, offering empirical evidence to the theoretical frameworks proposed by Bouchaud and Cont [4] and Li et al. [33,34]. The relationship between Market Temperature and liquidity measures also corresponds with Holden et al.’s [21], Aitken and Comerton-Forde’s [22] research on liquidity dimensions, validating the significance of thermodynamic measures in understanding market participant behavior.

## 8. Conclusions

The application of thermodynamics to the limit order book (LOB) represents a significant advancement in the field of econophysics, providing an innovative understanding of market dynamics. This study demonstrates the potential of applying thermodynamic principles to analyze and interpret the dynamics of the LOB. The empirical analysis of high-frequency trading data from the cryptocurrency market reveals the intricate interplay of order activities and market participant behavior in the LOB and their impact on order book dynamics. The findings suggest that Market Temperature can serve as a reliable indicator of market liquidity, while Market Entropy provides insights into the instantaneous volatility of the price dynamics in high-frequency trading markets.

Despite its novel approach, this study encounters several limitations and demands further investigation. First, the LOB is occasionally elusive with potential market manipulation activities. The efficacy of thermodynamic measures in identifying and mitigating the influence of such activities while studying the dynamics in the LOB remains for discoveries. Second, in the stability of links between Market Temperature and liquidity measures, for instance, the inconsistent correlation between the Market Temperature and match volume across datasets suggests variability in these relationships under different market conditions. This highlights the demand for further research to understand the underlying mechanisms driving these dynamics. Future studies should explore how different market states or regimes influence the relationship between thermodynamic measures and traditional financial metrics. Additionally, the datasets are taken in a specific time frame from a single cryptocurrency exchange. As a result, expanding the scope beyond Bitcoin to validate other cryptocurrencies and different financial asset classes such as stocks and commodities could provide a more comprehensive understanding of these dynamics. This could involve developing more characterized models or algorithms that account for varying market conditions, further enhancing the predictive capability and applicability of these thermodynamic concepts in the LOB.

In conclusion, this interdisciplinary approach combining physics and financial economics stimulates the potential of econophysics in modeling the complex systems in financial markets, offering a deep understanding of the complexities intrinsic in the market microstructure. The insights gained from this study are invaluable in shaping the future landscape of quantitative finance and econophysics.

## Figures and Tables

**Table 1:** Nature of the datasets.

Dataset	Start Time	End Time	Datapoints of Event Data	Datapoints of Snapshot Data
Dataset 1	2023-11-16 09:05:17.480629	09:51:28.912841	1,485,331	337,072
Dataset 2	2023-12-08 09:20:27.70310	10:25:15.618361	1,453,126	991,850

**Table 2:** Statistics of the datasets.

Dataset	Data Type	Mean	Median	Min	Max	St. Dev	Skew	Kurtosis
Dataset 1	event data (quote price)	37,292.31	37,344.12	1.00	125,000.00	1160.01	−23.49	1125.27
Dataset 1	snapshot data (mid price)	37,342.95	37,346.18	37,201.63	37,460.99	65.08	−0.18	−1.02
Dataset 2	event data (quote price)	43,145.17	43,236.34	1.00	476,631.00	1763.86	35.75	12,144.52
Dataset 2	snapshot data (mid price)	43,205.31	43,205.71	43,087.28	43,301.72	53.49	0.179	−1.23

**Table 3:** The regression analysis for Delta Entropy (ΔS) vs. Instantaneous Volatility (σ).

Dataset	$R^{2}$	F-Statistic	Prob (F-Statistic)	Omnibus	Prob (Omnibus)	Skew	Kurtosis	Durbin–Watson	Jarque–Bera (JB)	Prob (JB)
Dataset 1	0.414	4640	0	953.562	0	0.528	7.627	0.034	6156.622	0
Dataset 2	0.566	1300	$8.37\times 10^{-183}$	141.995	0	0.916	4.542	0.070	238.374	$1.73\times 10^{-52}$

**Table 4:** The fitted model for Delta Entropy (ΔS) vs. Local Volatility ($\sigma_{LV}$).

The Fitted Model	Coefficient	Standard Error	*t*	P≥|t|	[2.5%,	97.5%]
$\alpha_{1}$	0.2552	0.008	30.544	0	0.239	0.272
$\beta_{1}$	0.0009	$1.27\times 10^{-5}$	68.114	0	0.001	0.001
$\alpha_{1}$	0.1178	0.019	6.359	0	0.081	0.154
$\beta_{2}$	0.001	$2.71\times 10^{-5}$	36.051	0	0.001	0.001

## Data Availability

The datasets used or analyzed during the current study are available from the corresponding author on reasonable request.
